# Opening a Window on Attention: Adjuvant Therapies for Inflammatory Bowel Disease

**DOI:** 10.1155/2020/7397523

**Published:** 2020-08-12

**Authors:** Qiyue Wang, Shuyi Mi, Zixuan Yu, Qiyi Li, Jiacai Lei

**Affiliations:** ^1^Department of Gastroenterology, Hangzhou Da Jiangdong Hospital, Hangzhou, Zhejiang Province, China; ^2^School of Medicine, Zhejiang University City College, Hangzhou, Zhejiang Province, China

## Abstract

Inflammatory bowel disease (IBD), most commonly known as Crohn's disease (CD) and ulcerative disease (UC), is a chronic and relapsing intestinal disease which cannot be cured completely. The prevalence of IBD in Europe and in North America has increased over the past 20 years. As most IBD patients are young at onset, their quality of life (QOL) can be influenced to varying degrees. Thus, current treatment goals are typically focused on preventing complications, including maintaining clinical remission and improving the QOL. Adjuvant therapies have been widely concerned as an effective treatment in alleviating IBD symptoms, including dietary intervention, traditional Chinese medicine, smoking, alcohol, and physical activities. This review focuses on different ancillary therapies for IBD treatments, in particular the mechanism of reducing inflammation based on the actual data from research studies. Moreover, comparing the latest data, this review also presented potential future prospect for adjuvant therapies.

## 1. Introduction

Inflammatory bowel disease (IBD) is a chronic, immune-mediated disease mainly consisting of two diseases, Crohn's disease (CD) and ulcerative colitis (UC) [1]. CD is active in any part of the gastrointestinal tract from the mouth to the anus, while UC is restricted to the colonic mucosa [2]. The etiology and pathology of IBD remain largely unknown, but a complicated interplay of genetic, environmental, immunological, and lifestyle factors has been associated with this condition [3, 4]. Clinical symptoms of IBD involved but not limited multiple organs and systems throughout the body, such as nodular erythema, osteoporosis, and IBD-related arthropathy.

The current medical treatment in IBD is available for IBD patients: drug-based therapies such as nonsteroidal anti-inflammatory drugs (sulfasalazine and masalazine), biological therapies such as anti-TNF-*α* antibodies, and immunomodulators such as methotrexate. Meanwhile, surgical management is used to control the progression of severe disease. Unfortunately, these drugs also cause adverse effects in IBD patients, and the high disease cost with a long treatment process has brought economic burden for patients. For example, biological therapies such as anti-TNF-*α* may increase the risk of infection, and the costs of these drugs vary from 3,900 to 6,000 US dollars [5]. Therefore, it is not surprising that patients seek complementary and adjuvant therapies in IBD.

In this review, we focus on rational dietary structures, Chinese traditional medicine, suitable smoking, alcohol, and physical activities. Rational dietary structure has been speculated to be a pivotal factor in the pathogenesis of IBD and may be important in managing disease symptoms. IBD patients choose various dietary strategies to minimize gastrointestinal distress and improve overall health. Dietary products are the most common antigens in the intestine, altering the composition of the intestinal flora and changing the permeability of gastrointestinal tract [6]. Dietary interventions may not be appropriate alternatives to conventional medical therapy, but are effective complementary approaches for IBD treatment. In this review, we focus on different dietary components which are reported to benefit patients' symptoms.

The popularity of Chinese traditional medicine (CAM) has been systematically increasing among various complementary and adjuvant medicine approaches for the treatment of gastrointestinal disorders, mainly because it is natural and effective. CAM products range from homeopathy, herbal medicine, acupuncture, and moxibustion. Although the CAM is often of yet unknown efficacy and mechanism, the induction and maintenance of disease remission in UC and CD have been investigated.

Over the last few years, it is controversial whether IBD patients should quit smoking and alcohol. Cigarette smoking has been shown to worsen disease activity in CD, while in UC, smoking decreases the extent of disease [7]. Another important factor is alcohol; alcohol has been previously shown to be associated with worsening GI symptoms, but a case-control study illustrated that moderate red wine consumption by patients is linked with a lower risk in IBD [8].

Physical activity is one candidate complementary intervention that is of potential benefit in various chronic diseases. Previous studies showed that IBD patients perceive physical activities as a helpful management in reducing symptoms and complications of IBD [9]. There are physiologic benefits to physical activity such as improved bone density, decreased incident of colitis associated colorectal cancer, and the prevention of obesity [10]. Swimming, walking, and Chinese martial arts such as Qigong and Tai chi have been shown to improve the QOL, balance internally for healing, and improve bone density [11]. We will summarize the role of different physical activities in the development and course of IBDs.

## 2. Dietary

Diet is closely linked with IBD, especially in Western diet. Currently, dietary interventions have been studied in IBD to alleviate active disease and maintain remission. The Mediterranean diet pattern has been shown to be protective in IBD, as the incidence of IBD in the south of Europe is lower than in the northern Europe. Some of the components in the Mediterranean diet pattern such as olive oil, fish oil, fruits, and vegetables have been shown to be efficacious and patient-friendly.

Epidemiological studies illustrated that the intake of a higher ratio of *n* − 6 polyunsaturated fatty acids (*n* − 6 PUFAs) and a lower ratio of *n* − 3 polyunsaturated fatty acids (*n* − 3 PUFAs) was associated with an increased risk of developing IBD [12, 13]. Researches to date have demonstrated that *n* − 3 PUFAs may offer a promising approach to improving dysbacteriosis, reducing the likelihood of relapse, and lowering the mortality of colitis [14, 15]. Moreover, their protective effect in IBD is hypothesized to be derived from the balance in the ratio of *n* − 6/*n* − 3 PUFAs (0.65 or higher) [16]. The hypothesized machanism underlying the anti-inflammatory effect is to release proinflammatory mediators, reduce free-radical generation, and platelet activating factor formation, all of which are increased in IBD [17, 18].

Currently, fish peptide seems to have tissue reparative properties based on several studies in rodents and human. Salmon fillets contain *n* − 3 PUFAs, and marine collagen peptide has an anti-oxidative effect. A previous study showed that a regular intake of salmon in patients with UC is beneficial based on the improved simple clinical colitis activity index (SCCAI) and anti-inflammatory fatty-acid index (AIFAI) [19]. The combination of fish peptides and fish oil diet was more efficient than pure fish oil in an animal models study [20]. Compared with the pure fish oil, adding the fish peptides diet effectively reduces the production of proinflammatory cytokines and increases the level of PGE_3_ in plasma [20]. Additionally, other dietary peptides also have demonstrated an anti-inflammatory effect in IBD animal models (Machbank et al.) that dish hydrolysate was suggested to have an intestinal protective effect in mice [21]. Based on these observations, the fish peptide diet may be an effective way to maintain remission IBD.

Dietary fiber is more commonly used as a supplement for the management of IBD. Rational intake of fiber may reduce CD risk, especially that which originated from fruits [22]. Fibers from fruit have anti-inflammatory properties and positive modulation of the intestinal microbiota [23]. Fibers fermented by bacteria in the colon produce the short-chain fatty acids inhibiting the activation of transcription of proinflammatory mediators [24]. According to a case-control study, high fiber intake respondents were 41 percent less likely to develop CD than those with low fiber intake (median 24.3 g/day) [25]. However, fiber intake is controversial. Scientists found that the risk of fiber was >15 g/day, while Thornton showed there was no difference between patients and controls [12]. The future study should pay more attention to the amount of fiber and IBD.

Vitamin D is a group of fat-soluble vitamins which plays a key role in IBD treatments [26]. Recent studies have reported that Vitamin D was linked to the protection against infection and the control of the gut commensal microbial composition [27, 28]. Clinical trials have suggested a positive correlation between Vitamin D deficiency and IBD that nearly half of the patients had hypovitaminosis D [29–31]. Vitamin D deficiency may reduce the expression of a tight junction in the intestinal epithelium, decrease the clearance of colonic bacteria directly, and affect the gut barrier and immune system functions that impact the onset and progression of IBD [29–31]. To correct vitamin D deficiency, researchers found that, after ingestion of 1,200 IU vitamin D3 daily for 3 months and 50,000 IU vitamin D2 weekly for 6 weeks, the vitamin D status was significantly improved and 78% of the patients' CD activity index (CDAI) had a drop of less than 70 points compared with placebo among children and adolescents with IBD [32]. Therefore, in CD, the effective dose of vitamin D3 was determined at 5000 IU/d [33].

## 3. Traditional Chinese Medicine

In recent years, traditional Chinese medicine (TCM), including herbal medicine, acupuncture, and hemopathy has been commonly used among IBD patients from all age groups. It is estimated that the percentage of IBD patients using TCM in North America at 21% might increase up to even 60% [34]. Herbal medicine is the most common TCM modality with lower cost and higher efficiency. TCM herbal enema has been proved to be the most efficient method for therapies because of the regulation of immune responses in the colon mucosa [35]. The mechanism of TCM in IBD includes improving anti-inflammatory activities and reducing the level of proinflammatory cytokines [35].

Yun Nan Bai Yao (YNBY) is a Chinese herbal remedy used for treating wounds for its hemostatic properties [36]. In the recent years, researchers showed that YNBY can effectively reduce the severity of experimental colitis by the immunosuppression and wound healing mechanisms. It was shown that YNBY significantly suppresses the growth of T lymphocytes and B lymphocytes, thus decreasing several proinflammatory cytokines such as TNF-*α* which were closely correlated with IBD [5]. A dose-dependent hemostatic effect was also revealed by researchers through rabbits models [36]. Therefore, for patients suffering from gastrointestinal bleeding, giving YNBY enterally may serve as an effective adjunctive therapy. YNBY is also capable of reducing intraoperative blood loss. The dosage of YNBY on CD is still unclear, and future studies should focus on the dosage and indications for IBD patients.

Tripterygium wilfordii Hook F (TWHF), aloe vera, and tormentil are TCM herbal remedies with anti-inflammatory activities. It has been shown that TWHF achieves the same level in preventing the postoperative recurrence of CD [37]. In the previous research, the relapsed patients in the TW- and mesalazine-treated groups were 18.2% versus 21.7% in 6 months and 45.5% versus 60.9% in 1 year. Moreover, the CDAI was decreased during the first 8 weeks and reached the minimum in week 10 [37]. It was also reported that 47% respondents' symptoms are alleviated through using aloe vera [37]. Tormetil extracts were suitable for chronic IBD patients, which was proved to be safe up to 3000 mg/day for 3 weeks with minor side effects [38].

Another herbal therapy such as Food Allergy Herbal Formula-2 (FAHF-2) which originated from Wu Mei Wan that has long been used in China to treat colitis may have the possibility to be served as a novel treatment of CD [39]. Composed of *Aconitum*, *Coptis*, ginger ginseng, *Cinnamomum*, *Angelica*, and *Ganoderma lucidum*, it was found to be able to inhibit both adaptive and innate immune proinflammatory cytokine responses in inflamed CD mucosa and valid in halting progression of colitis in a murine model, which indicated that FAHF-2 may be safe and effective for CD to maintain remission and avoid the need for pharmacologic escalation in therapy with medications that have potentially severe side effects [39, 40].

*C. longa* L. (turmeric) is a plant whose root segment is commonly used as a seasoning and in traditional Chinese medicine for thousands of years. Curcumin is the chief biologically active derivative of turmeric. In a series of in vitro and in vivo studies, curcumin has recently caught much attention for its anti-inflammatory characteristic. Curcumin is capable of correcting abnormal immune response in IBD through decreasing proinflammatory cytokines synthesis, downregulating the transcription of the proinflammatory gene by inhibiting NF-*κβ*, and upregulating antioxidant enzymes [41].

A placebo-controlled double-blind study proved that a proper combination of curcumin and mesalazine can effectively induce remission in mesalazine-tolerant UC patients (whose disease course fail to improve under maximum dose of mesalazine for 2 weeks). After standard add-on curcumin therapy (3 g/day, in capsules) for 1 month, 56% (14/25) of patients in the curcumin-treated group achieved clinical remission and 10/22 (45.4%) presented amelioration in endoscopical performance (evaluated by the endoscopic Mayo index subscore) while none achieved remission (0/22, 0%) in the placebo group [42].

Curcumin is nontoxic even at relatively high doses (with no known toxic side effects in humans up to doses of 12 g/day), and even pediatric patients with IBD are able to tolerate a curcumin dose up to 2 g twice/day and some of them demonstrate improvement in the disease course [43, 44].

Currently, popular anti-inflammatory therapy can lead to tremendous cost, while curcumin remedy is much more affordable with a price less than $5 per week. For its effectiveness, safety, and affordability, curcumin could be an ideal agent for curing IBD. However, it is hard to maintain therapeutic curcumin concentration pattern in human body for its rapid metabolism and dissatisfied biodistribution [43]. More effort should be made to determine a feasible method of administration, as well as improve its absorption and biodistribution before curcumin can fully benefit IBD patients.

## 4. Smoking

Smoking affects these CD and UC differently; many case studies suggest that smoking is a risk factor for CD while it tends to confer a protective effect against UC [45, 46].

Compared to never-smokers, the incidence and severity of UC are lower in smokers in many studies, and smoking exerts protective effects on both the development and the progression of UC [47, 48]. The relapse rate, hospitalization rates, and the need for oral steroids and the colectomy rates were found to be lower in current smokers rather than nonsmokers [49]. It was observed that high cigarette dose such as 20 cigarettes per day was correlated with less extensive colitis and lower treatment needs [50]. However, the protective role of smoking in UC sustains until 2–5 years after smoking cessation, and then, the risk goes up; interestingly, the risk paralleled past cumulative exposure with an increase in the disease activity and the need for hospital admission and major medical therapy [51, 52].

As for the influence of smoking on CD, most studies have suggested that current smokers have a higher risk of developing CD than those who have never smoked [53, 54]. Besides, previous data have demonstrated that smoking was associated more frequently with complicated disease such as penetrating intestinal complications and a higher relapse rate [48]. It was shown that patients with a high life time tobacco exposure (>150 cigarette years) and heavy smokers (>10 cigarettes/day) had small bowel disease more often than the patients with both lower life time exposure (<150 cigarette years) and smoking ≤10 cigarettes/day [55]. Actually, the increased risk of disease relapse is significantly over a threshold (15 cigarettes per day) [52]. In addition, compared with nonsmokers, the needs of steroids and immune suppressants rise in smokers [56–58]. The impact of cigarette smoking on CD is temporary, smoking cessation improves the course of the disease, and it has been estimated that after 2 years of smoking cessation, former smokers underwent a process similar to that of patients who have never smoked and the flare-up rate was decreased 65% [59, 60].

Notwithstanding, it has been noted that the use of snus was not associated to the development of either UC or CD, which underlines differences in combusted and noncombusted tobacco in the genesis of IBD [61, 62]. In addition, the fact that snus users have higher levels of the nicotine metabolite cotinine also implies that nicotine by itself may not be involved in the pathogenesis [62].

## 5. Alcohol

Alcohol is another potential trigger for flaring IBD since alcohol in different amount of consumptions affects the immune system and results in various imbalanced organs with inflammation [63–65]. Furthermore, alcohol consumption has an effect on gut permeability and plasma levels of gut-derived bacterial products such as lipopolysaccharides and peptidoglycans [66]. However, compared with never drinking, light drinking has a protective effect on the development of UC [67]. It stands to reason that hazardous alcohol intake (it is defined as more than 40 g of alcohol per day for men and 20 g for women) is detrimental for IBD patients while moderate red wine consumption lasted for a week is linked with a lower risk in IBD. The mechanism is the decrease in stool calprotectin which is known as an antioxidant with anti-inflammatory effects [68, 69]. A research showed that fecal stool calprotectin was decreased compared with baseline after 1 week of drinking in IBD patients [67]. Antioxidants with additional anti-inflammatory actions may benefit the treatment in IBD on account of the mechanism in inflammation caused by oxidative stress [70, 71]. Oxidative stress shows a definition of the imbalance between oxidants (reactive oxygen species and reactive hydrogen species) and antioxidants which is linked to chronic intestinal inflammation in the early stage of IBD [71]. Meanwhile, alcohol inhibits the immune system by reducing interleukin (IL)-12 and increasing interleukin (IL)-10 production which can affect the induction on Th1 or Th2 immune responses [69, 72, 73]. Moreover, autocrine IL-10 production can prevent maturation of dendritic cells and induce anergy in the T-cells responder which is an anti-inflammatory cytokine closely related to the immune system [74]. However, alcohol consumption was controversial nowadays. A report showed that although beer is beneficial for some people (though only a few), more than 55 percent of the participants reported worsening symptoms from drinking. Moreover, only about 5 percent patients was tolerant to wine, while more than 50 percent of the subjects showed an increase in red wine symptoms [75]. Therefore, the future study should focus more on the dosage and side effect of alcohol consumption.

## 6. Physical Activity

Physical activity may potentially play a role in alleviating symptoms related to extraintestinal manifestations of IBD [9]. Previous study showed patients with, at least, 27 metabolic equivalent task (MET) hours per week of physical activity have a 44% reduction in risk of developing Crohn's disease compared with those with <3 MET·h/wk [76]. Exercise can be beneficial for intestinal and extraintestinal manifestations of IBD that regular physical exercise could improve physiological health, maintain their weight, improve bone mineral density, and alleviate the anorexia caused by IBD [2, 77].

Low-to-moderate intensity physical activities have been proved to be safe and suitable that it was well tolerated by IBD patients especially those who were in remission and did note provoke subjective symptoms [78]. Moderate-intensity physical activity has beneficial effects on the gastrointestinal system. Patients with regular exercise during the previous 5 years may have lower chance of developing CD, especially if the exercise is performed daily. Additionally, it was also demonstrated that patients who performed exercise were less likely to develop the disease activity in UC [79]. Several possible mechanisms may explain the anti-inflammatory influence of physical activities, including releasing interleukin-6 (IL-6) from skeletal muscle, inhibiting the TNF production, and stimulating the release of IL-10 [80, 81]. However, extreme exercise may contribute to intestinal inflammation by the means of increasing the number of CD4 and CD8 lymphocytes, natural killer cells, and the level of reactive oxygen species [79]. It seems that the effect of physical activity depends on the intensity and duration of the physical activity.

Some of the aspects were also observed at experimental conditions because moderate voluntary treadmill exercise could significantly accelerate the healing of colitis in IBD. A recent study by Cook et al. showed that 30 sessions of forced treadmill exercise training exacerbated inflammation in dextran sodium sulfate-induced colitis, proved by excessive diarrhea episodes and increased animal mortality [82]. In a previous study, a long-term physical activity of 6-week running attenuated the colonic TNF-*α* protein content, indicating the anti-inflammatory effect of physical exercise [2, 83]. According to the previous rodent studies, exercise could downregulate the expression of interleukin 1-*β* (IL-1*β*) and TNF-*α* in both colonic mucosa and plasma. Moreover, it was demonstrated that leptin levels were significantly decreased, which diminished the severity of colonic damage mediated, and exerted an anti-inflammatory effect on an inflamed colon [84].

Swimming and cycling are two effective aerobic exercises that can be beneficial with fewer gastrointestinal symptoms by the means of inflammatory modulation and apoptosis [10, 78]. In addition, walking 20–30 min at 60% of maximal heart rate 3 days per week along with resistance training 2-3 times per week is advocated in many studies, and it may have the potential to decrease the risk of active disease at six months [79, 85, 86].

Qigong and Tai Chi are traditional Chinese physical activities which coordinate the body and mind. These approaches have been shown to be effective in reducing symptoms such as fatigue, depression, and pain and improving QOL in a way of moderate exercise [11]. Qigong could improve immune functions and reduce inflammation profiles such as proinflammatory cytokines (TNF-*α*) which were correlated to IBD. Lymphocytes, thyroid-stimulating hormone, and IgG were found to be modulated in response to practicing Tai Chi. Researchers demonstrated that a 30-minute weekly session of Qigong with a duration of 8 weeks is recommended. Moderate Tai Chi and Qigong may enhance physiological and psychological function, and the future study may concentrate more on the suitable amount of exercise in Tai chi and Qigong [87].

## 7. Discussion

Considering the past few years, the adjuvant treatments for IBD have become increasingly important to better the immunity and inflammation in the intestine. More and more investigations illuminated the significance of diet, traditional Chinese medicines, and proper exercise, which are capable of taking the edge off withdrawal symptoms in IBD. Furthermore, smoking and alcohol mainly act as two environmental factors in adjuvant treatments [88].

Though unable to replace the conventional IBD therapies totally, adjuvant treatment, still plays an instrumental supplement role in treating the disease. Some patient-friendly components of the Mediterranean diet have been proven to affect the intestinal barrier and immune system function, thus affecting the progress of IBD. Traditional Chinese medicine can effectively reduce inflammatory cytokines and alleviate IBD. Moderate exercise such as Qigong, Tai chi, swimming, and walking are effective treatments in reducing the risk of pain and complications with less expenditure [9]. Moreover, achieving smoking cessation is also an important goal of IBD treatment for the beneficial effects of smoking on disease are offset by the harmful effects of tobacco on the respiratory and cardiovascular system [89, 90], whereas reasonable alcohol drinking benefits CD patients by not affecting the immune system [68, 69].

However, as a novel treatment for CD, several weaknesses have been limited by the application of adjuvant treatments. It is of ambiguity whether adjuvant treatment is suitable or curable for all types of IBD patients. For example, some changes associated with diet on the maintenance of IBD remission are ambiguous, and the affecting dosage for IBD of each beneficial dietary component is unknown. For herbal medicines, there has been no specific research to confirm a particular dose or prescription has significant effects on patients. In addition, although previous studies have vitrified the benefits of exercise in IBD treatment, it has not been defined as the most appropriate adjuvant for all types of IBD, especially in Qigong and Tai chi. For UC patients, smoking was found to be more conducive while it has strong linkage with pernicious diseases all the time. So, conducting high-quality clinical trials with appropriate blinding and large number of patients is necessary to obtain more conclusive results on the curative effect of adjuvant treatment in IBD.

This paper systematically expounds the significance and different factors of IBD adjuvant treatments within the field of comprehensive treatments in IBD. Adjuvant treatment is most approbated among the public nowadays for its relatively low cost and minor side effects compared with traditional remedies. Moreover, this review also aims to draw the attention of the public to engage patients in a discussion of adjuvant treatment and underline their role as a complement to conventional IBD therapies. Physicians are urged to explore the use of adjuvant treatment and provide appropriate information and guidance to patients in order to develop high-quality care for patients with IBD.

## Data Availability

The data used to support the findings of this study are available from the corresponding author upon request.

## Supplementary Materials

Supplementary Materials Supplementary materials: the supplementary material file is a figure describing the mechanism and effect of physical activities that could alleviate gastrointestinal symptoms and improve patients' quality of life. Physical activities such as swimming, walking, Tai Chi, and Qigong may induce anti-inflammatory modulation in releasing the interleukin-6 (IL-6), interleukin-10 (IL-10), lymphocyte, and Immunoglobulin G (IgG). Moreover, the expression of interleukin 1-*β* (IL-1*β*) and the TNF-*α* protein content would be downregulated due to moderate physical activities. In addition, the physiological health would be better and the hospitalization time could be shortened through physical activities.
