# Supplementary material for: Opening a Window on Attention: Adjuvant Therapies for Inflammatory Bowel Disease
Source: Can J Gastroenterol Hepatol. 2020 Aug 12;2020:7397523. doi: 10.1155/2020/7397523 (PMC7441453; doi:10.1155/2020/7397523)
Supplement: Supplementary Materials — the supplementary material file is a figure describing the mechanism and effect of physical activities that could alleviate gastrointestinal symptoms and improve patients' quality of life. Physical activities such as swimming, walking, Tai Chi, and Qigong may induce anti-inflammatory modulation in releasing the interleukin-6 (IL-6), interleukin-10 (IL-10), lymphocyte, and Immunoglobulin G (IgG). Moreover, the expression of interleukin 1-β (IL-1β) and the TNF-α protein content would be downregulated due to moderate physical activities. In addition, the physiological health would be better and the hospitalization time could be shortened through physical activities. [file 7397523.f1.docx]

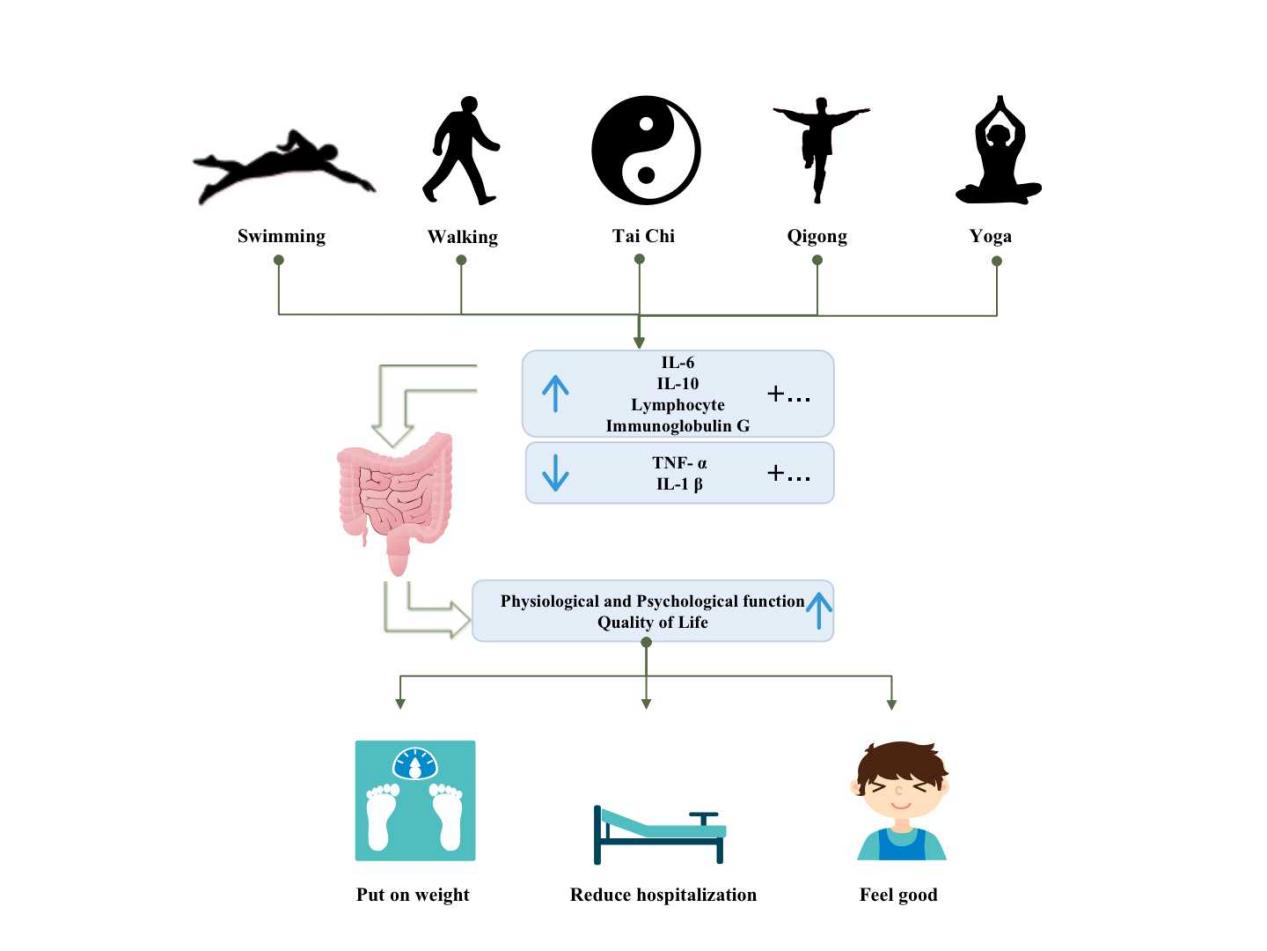


Figure S1. Mechanism and effect of Physical Activities. Physical activities alleviate the gastrointestinal symptoms by down-regulating the expression of IL-1β and TNF-α protein content, and inducing an anti-inflammatory modulation in releasing IL-6, IL-10, lymphocyte and Immunoglobulin G(IgG).
